# The Effect of Humidity and the Role of Visual Cues During Feeding on Green/Brown Color Polyphenism in *Locusta migratoria*

**DOI:** 10.1002/arch.70044

**Published:** 2025-02-25

**Authors:** Keiryu Hirota, Ryo Watanabe, Ryohei Sugahara

**Affiliations:** ^1^ Faculty of Agriculture and Life Science Hirosaki University Hirosaki Aomori Japan

**Keywords:** body‐color polyphenism, food availability period, green/brown polyphenism, humidity, light cycle, migratory locust

## Abstract

The regulation of body color in locusts comprises phase polyphenism, green/brown polyphenism, and homochromy. Humidity was believed to control green/brown color polyphenism in locust species. However, recent findings indicate that humidity has minimal influence on this polyphenism in *Schistocerca gregaria* nymphs reared in isolation. This study investigated the effects of humidity and visual stimuli on *Locusta migratoria* nymphs reared in isolation. When *L. migratoria* nymphs were fed either *Bromus catharticus* or *Dactylis glomerata* leaves only during the dark period, the proportions of green nymphs at the 5th instar were comparable. Increased humidity (through addition of moist cotton) induced more green morphs under a 16‐h dark/8‐h light cycle, suggesting that humidity is involved in green/brown polyphenism in *L. migratoria*. However, these nymphs were not sensitive to humidity regarding this polyphenism under a 23‐h dark/1‐h light cycle, suggesting that sensitivity is related to visual cues. The daily overlap between food availability and light periods for 12 h resulted in a higher proportion of green morphs than a daily overlap for only 4 h, suggesting that such an overlap affects green/brown polyphenism in this species. Although we were unable to assess the effect of the total food availability period on polyphenism in this study, we confirmed that at least (1) humidity and (2) visual cues during feeding are associated with green/brown polyphenism in *L. migratoria*. These regulations may have ecological significance for this species, allowing them to phenotypically adjust to the seasonal and spatial variation in their environment, increasing their chances of survival.

## Summary

- Higher humidity often induces more green morphs in *Locusta migratoria* nymphs.
- Green/brown polyphenism is not always influenced by humidity.
- Longer overlap of the food availability and the light periods induces more green morphs.

## Graphical abstract

Green/brown polyphenism occurs in various insects including *Locusta migratoria*. Our study revealed that higher humidity induced more green morphs under a 16‐h dark/8‐h light cycle. Furthermore, the daily overlap between food availability and light periods affected the expression of green/brown polyphenism in this species.

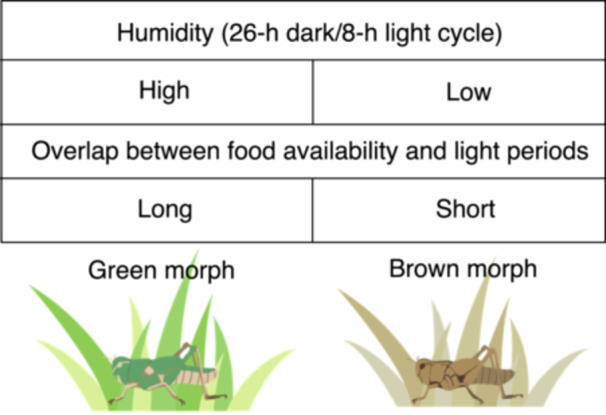

## 1 Introduction

Some animals can reversibly change their body color in response to internal and external environmental stimuli. This phenomenon plays a crucial role in thermoregulation, ultraviolet protection, crypsis, aposematism, and intraspecific communication in arthropods (Chapman 1998; Umbers et al. 2014). Reversible color changes can be categorized as morphological or physiological (Goda and Kuriyama 2021; Umbers et al. 2014): morphological color changes involve alterations in the morphology and density of chromatophores, which are specialized pigment‐containing and light‐reflecting cells found in various animals (Sugimoto 2002), whereas physiological color changes arise from rapid motile responses of chromatophores, manifesting colors more quickly (milliseconds to hours) than morphological changes (days to months) (Umbers et al. 2014). Reversible color changes observed in grasshoppers and locusts are primarily morphological (Filshie et al. 1975; Umbers et al. 2014). However, the chameleon grasshopper *Kosciuscola tristis* (Sjöstedt) has been reported to exhibit physiological color changes from black to turquoise in response to increasing temperatures (Key and Day 1954a, 1954b; Umbers 2011).

Acridids display three main types of morphological color polyphenism: (1) phase polyphenism, (2) green/brown polyphenism, and (3) homochromy (Faure 1932; Rowell 1972). The last instar nymphs of the migratory locust *Locusta migratoria* (Linnaeus) exhibit all three polyphenisms in response to environmental conditions (Pener and Simpson 2009; Rowell 1972; Uvarov 1966). The regulatory model for body coloration in this locust is widely accepted as a hierarchical pathway (Pener 1991; Pener and Simpson 2009), where phase polyphenism sits at the top of the hierarchy. Nymphs at high population densities display dirty orange with black patterns, whereas those at low densities (solitarious nymphs) exhibit non‐contrasting coloration, either uniform or with faint patterns (Uvarov 1966). The coloration of solitarious nymphs is controlled by the second level in the hierarchy, green/brown polyphenism, in which high humidity conditions lead to green morphs and low humidity conditions lead to a non‐green uniform coloration (Faure 1932). The body color of the non‐green nymphs is regulated by the lowest level in the hierarchy, homochromy; the nymphs adjust their body color to match the substrate color of the habitat (Hertz and Imms 1937; Rowell 1972). Thus, *L. migratoria* last instar nymphs can develop a variety of body colors in response to environmental factors.

The regulation of body coloration in the desert locust *Schistocerca gregaria* (Forskål) has been explained by a model similar to that of *L. migratoria*, except for the lack of homochromy (Pener and Simpson 2009). However, Tanaka et al. (2012) reported that *S. gregaria* nymphs display homochromy. More importantly, they demonstrated that humidity had little effect on green/brown polyphenism in this species (Tanaka et al. 2010, 2012). In contrast, Hunter‐Jones (1962) reported that green/brown polyphenism is substantially influenced by humidity in *S. gregaria* nymphs. It is now believed that humidity possibly contributes to the expression of green coloration in these nymphs indirectly by maintaining the green color of the leaves, which stimulates locust vision (Tanaka et al. 2012, 2016). In Tanaka's experiment, the effect of the discoloration of grass leaves on visual stimuli was eliminated by supplying the leaves only during the dark period (Tanaka et al. 2012).

*S. gregaria* nymphs reared in isolation in cups covered with yellow–green paper tend to develop greener coloration than that developed by nymphs reared in isolation in cups covered with other colored papers, despite maintaining consistent humidity levels inside the cup (Tanaka et al. 2012, 2016). This suggests that green/brown polyphenism in this species is influenced more by visual information about the surrounding color than by humidity. A similar re‐examination of the effects of humidity on *L. migratoria* nymphs has not yet been reported. Thus, the role of humidity and visual information in regulating green/brown polyphenism in *L. migratoria* requires further investigation. In the present study, we investigated the factors that induce *L. migratoria* nymphs reared in isolation to become green by eliminating visual stimuli from grass leaves. We designed different light cycles, 23‐h darkness/1‐h light, 16‐h darkness/8‐h light, 8‐h darkness/16‐h light, and 12‐h darkness/12‐h light, to modulate the exposure time of test nymphs to the colors of the feeding grass and yellow–green paper. After several experiments, we found that the overlapping time of the food availability period (FAP) and light duration might play an important role in inducing green body color in *L. migratoria* nymphs. In other words, the overlap between the FAP and the light period, which was designed in our experiment to create artificial extreme conditions, might inadvertently mimic a realistic situation. Therefore, we further verified the role of visual information during feeding.

## 2 Materials and Methods

### 2.1 Insects and Container

**Figure 1:**
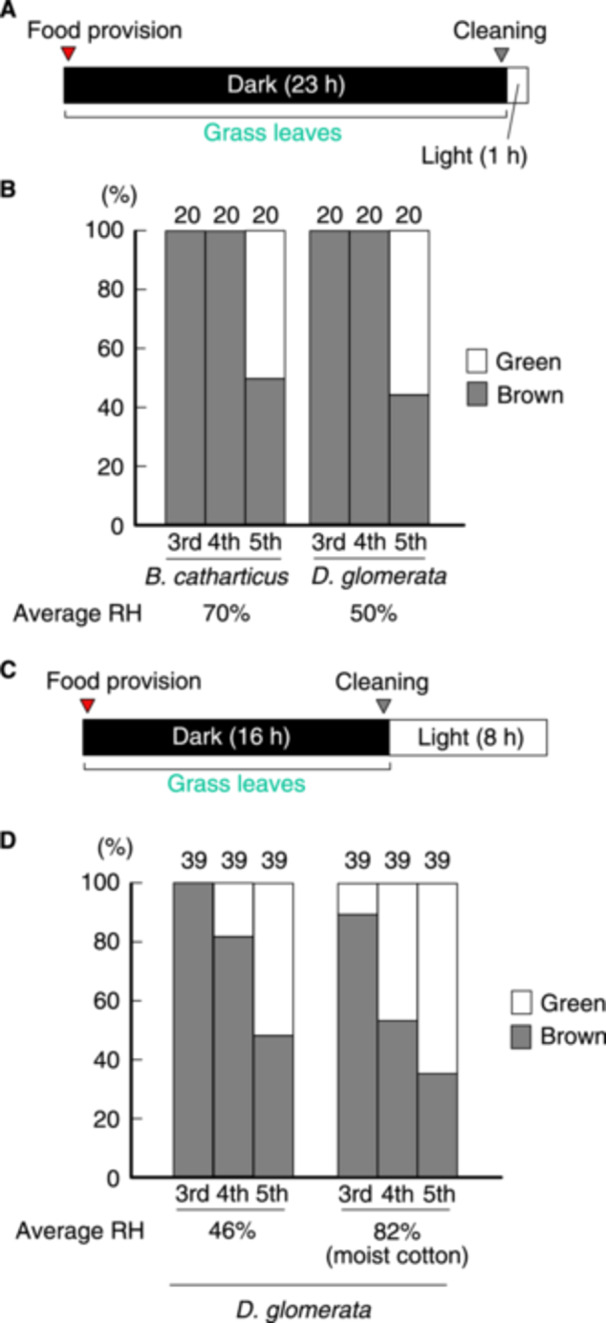
Proportions of green and brown morphs of *L. migratoria* that were fed during the dark period. A schematic of the rearing schedules within the daily light cycle is provided (A, C). Cut grass leaves were supplied just after the lights were turned off, and the cups were cleaned just before the lights were turned on. Proportions of green and brown morphs at specific instars were calculated (B, D).

**Figure 2:**
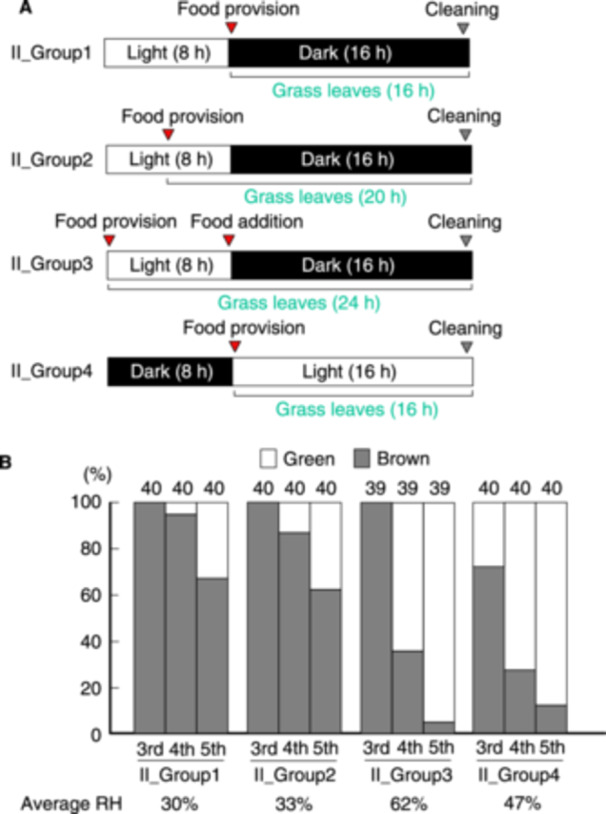
Proportions of green and brown morphs of *L. migratoria* that were fed for 16, 20, and 24 h under an 8:16 light:dark cycle. A schematic of the daily light cycle rearing schedules is shown (A). Grass was supplied just after the lights were turned off (*II_group 1*) or just after the lights were turned on (*II_group 4*). Cups were cleaned just before the lights were turned on (*II_groups 1* to *3*) or just before the lights were turned off (*II_group 4*). Proportions of green and brown morphs at specific instars were calculated (B).

**Figure 3:**
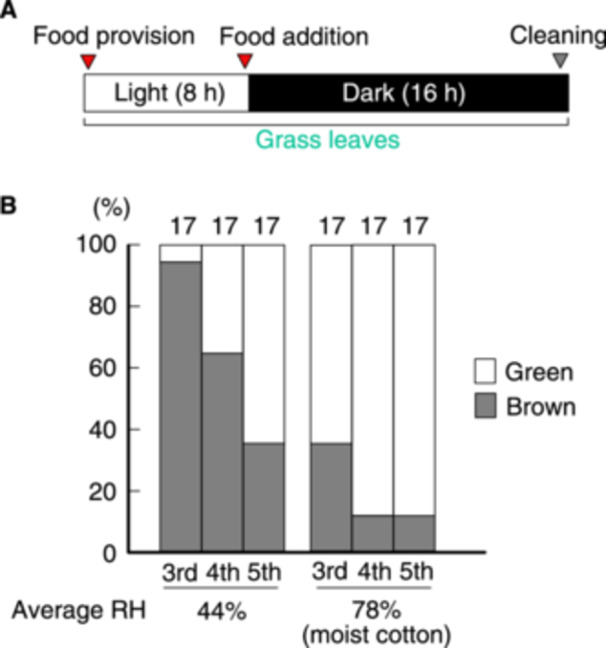
Proportions of green and brown morphs of *L. migratoria* fed for 24 h at high and low relative humidity. A schematic of the daily light cycle rearing schedule is provided (A). Food provision and cup cleaning were conducted just before the lights were turned on. Proportions of green and brown morphs at specific instars were calculated (B).

**Figure 4:**
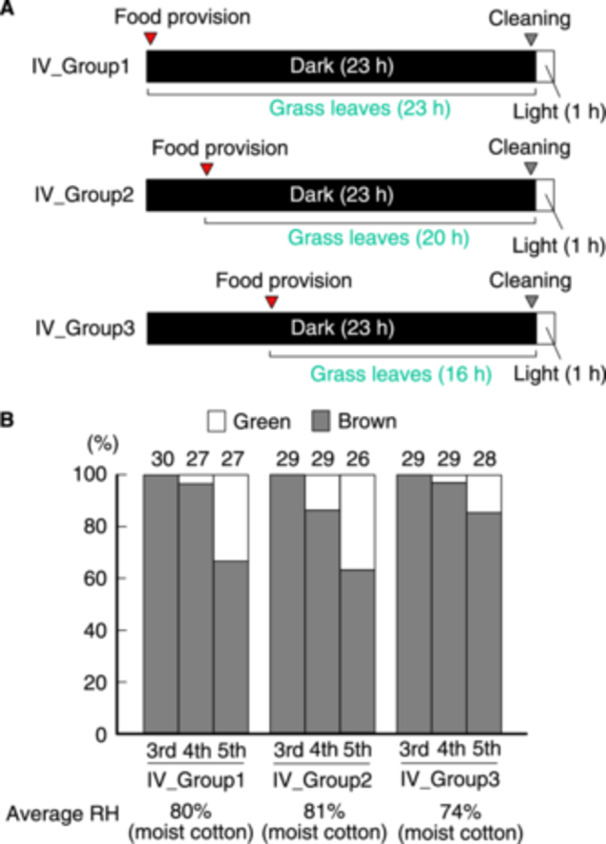
Proportions of green and brown morphs of *L. migratoria* fed for 23, 20, and 16 h under a 23‐h darkness/1‐h light cycle at high relative humidity. A schematic of the rearing schedules within the daily light cycle is displayed (A). Grass was provided just after the lights were turned off (*IV_group 1*), and the cups were cleaned just before the lights were turned on (*IV_groups 1* to *3*). Proportions of green and brown morphs at specific instars were calculated (B).

**Figure 5:**
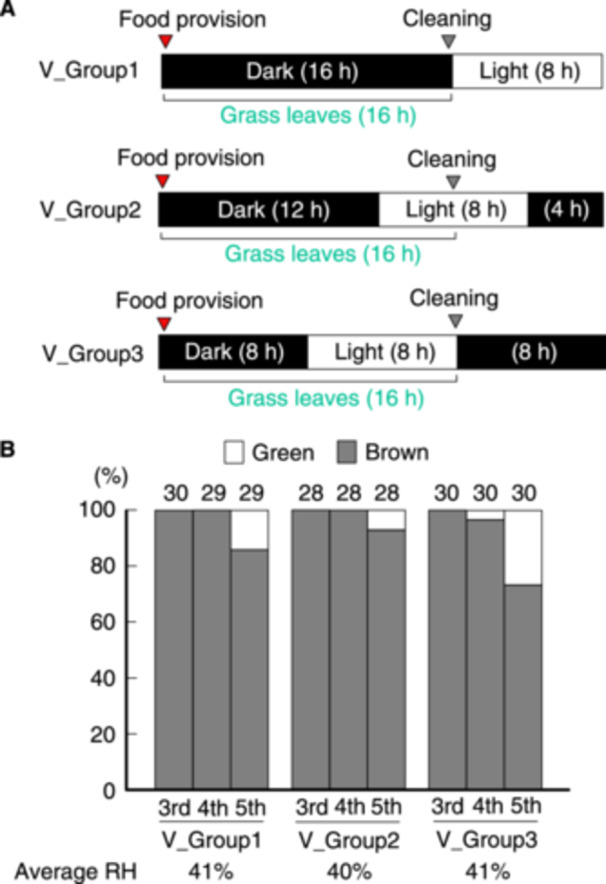
Proportions of green and brown morphs of *L. migratoria* fed for 0, 4, and 8 h during the light period under a 16‐h darkness/8‐h light cycle. A schematic of the rearing schedules within the daily light cycle is displayed (A). Grass was provided just after the lights were turned off (*V_group 1*), and the cups were cleaned just before the lights were turned on (*V_group 1*) or turned off (*V_group 3*). Proportions of green and brown morphs at specific instars were calculated (B).

**Figure 6:**
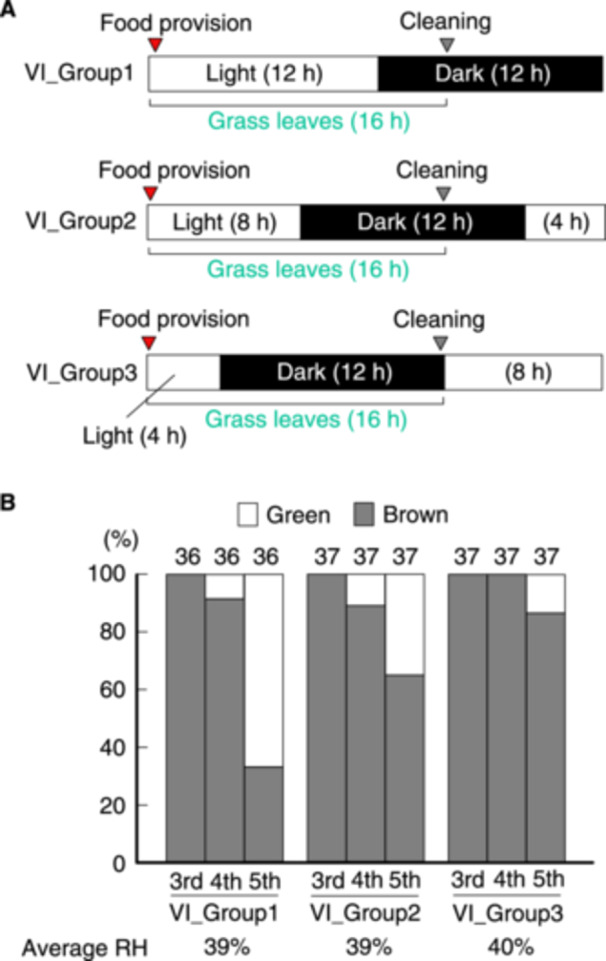
Proportions of green and brown morphs of *L. migratoria* fed for 12, 8, and 4 h during the light period under a 12‐h darkness/12‐h light cycle. A schematic of the rearing schedules within the daily light cycle is displayed (A). Grass was provided just after the lights were turned on (*VI_group 1*), and the cups were cleaned just before the lights were turned on (*VI_group 3*). Proportions of green and brown morphs at specific instars were calculated (B).

*L. migratoria* nymphs of the Minami‐Daito strain, derived from individuals collected on the Minami‐Daito Island, Okinawa (25.8° N, 131.2° E), were maintained under crowded conditions (gregarious phase) and used in this study (Sugahara et al. 2016). The locusts were fed grass leaves of *Bromus catharticus* (Vahl) (Poales: Poaceae) unless otherwise specified. *Dactylis glomerata* (Linnaeus) (Poales: Poaceae) leaves were also used as food (Figure 1). In our preliminary experiment, we found that 8 h of fasting in 1st instar nymphs resulted in high mortality rates during nymphal growth (Table S1). Because starvation tolerance increases with the nymphal instar stage in *L. migratoria* (Uvarov 1966), newly ecdysed 2nd instar nymphs (day 0) were used in this study. Hatchlings used in Figures 1, 2, 3, 4, 5, and 6A were obtained from the same egg pods within each respective experiment, and all groups within each experiment were reared concurrently. Hatchlings were supplied daily with freshly cut grass leaves (1–2 cm long) as food. They were individually reared in Petri dishes (diameter, 9 cm; height, 1.5 cm), the walls of which were covered with yellow–green vinyl tape (Sugahara et al. 2015; Tanaka and Nishide 2012). Day 0 2nd instar nymphs were transferred to transparent plastic cups (bottom diameter, 8 cm; lid diameter, 9 cm; height, 2.5 cm) with a perforated lid with four holes (diameter, 2 mm) for ventilation (Figure S2). The nymphs were visually isolated inside the cups, which had their bottom and inner walls covered with yellow–green paper (Daio Paper Products Co., Shizuoka, Japan; Figure S1B) (Tanaka and Nishide 2012). Although the 5th instar is the last nymphal stage in gregarious *L. migaratoria*, 6th instar nymphs are observed in less than 10% of females when the locusts are reared in isolation (Sugahara et al. 2015). In the present study, these nymphs were analyzed during their 5th (penultimate) instar.

### 2.2 Rearing Test Nymphs

Food provision and cleaning were performed at designated times, as illustrated in the corresponding figures. Pieces of cut grass leaves, sufficient for one nymph, were inserted into each container through a gap in the lid (Figure S1A). While cleaning the containers, each nymph was transferred to a plastic bag (26 × 38 cm) to avoid direct handling. Food provision and cleaning during the dark period were conducted under red light‐emitting diodes (TornadoACE 625 nm, Bio Medical Science Co., Tokyo, Japan) in the dark rooms and four growth chambers (MLR‐351G, Sanyo, Tokyo, Japan; MLR‐350, Sanyo; LJ‐411PDF‐S, Nippon Medical & Chemical Instruments Co., Osaka, Japan; CL‐301, Tomy, Tokyo, Japan) (Figure S1C). All test nymphs were reared at a constant temperature of 30°C in growth chambers. The positions of the shelves in the growth chambers were rotated daily to reduce bias.

### 2.3 Experimental Design

The effects of environmental conditions on green/brown polyphenism were examined by monitoring the morph transition of brown second‐instar nymphs to green morphs during the third, fourth, and fifth instars when reared in transparent cups covered with yellow–green paper. Environmental conditions were controlled by the presence of moist cotton balls, the timing of food provision and cup cleaning, and the regulation of the light/dark cycle. We initially prioritized designing a rearing schedule that avoided overlap between the FAP and the light period to minimize the effect of grass color on green/brown polyphenism. However, in subsequent experiments, we compared various durations of overlap between the FAP and the light period to investigate the factors influencing the emergence of green morphs. The experiments conducted to evaluate the effect of humidity on green/brown polyphenism are presented in Figures 1 and 3. To explore factors, other than humidity, influencing the emergence of green morphs, we performed the experiment depicted in Figure 2. The rearing schedule depicted in Figure 4 was designed to assess the effect of the total daily FAP on polyphenism. Finally, the experiments presented in Figures 5 and 6 were conducted to determine whether the overlap between the FAP and the light period influences polyphenism.

### 2.4 Food Availability and Fasting Periods

FAP refers to the duration during which grass leaves are present in the rearing cup. During the FAP, nymphs were free to feed at any time. In contrast, in most experiments, the timing of food provision and cup cleaning did not overlap, resulting in periods of food deprivation, referred to as fasting.

In some experiments, the daily FAP was extended to 24 or 23 h, which could lead to a loss of appetite in the locusts, as grass leaves dry over time. In *II_group 3* of Figure 2A, a loss of appetite would have compromised one of the experimental aims, that is, assessing the effect of the total FAP. Accordingly, the nymphs in *II_group 3* were additionally provided with fresh grass just before the dark period. Meanwhile, moist conditions created by damp cotton balls maintain the freshness of the grass leaves even during longer FAPs. Thus, we conducted the experiment shown in Figure 4 under moist conditions to decrease the effect of grass drying in *IV_group 1*. However, in Figure 3, the moist conditions could affect the locusts' appetite by moistening the grass leaves compared to dry conditions, which was an unintended effect. To reduce this effect, the test nymphs were additionally provided with fresh grass just before the dark period (Figure 3A).

### 2.5 Modulation and Measurement of Humidity

To modulate humidity inside the rearing cup, moist or dry cotton balls were placed in the cup. A portion of the cotton ball was exposed and encased in a silicon‐coated paper (Microwavable cup No.6; Daiso Industries Co., Hiroshima, Japan) (Figure S1D). The paper was secured with yellow–green vinyl tape. The damp cotton balls were moistened daily during the cup cleaning process. Relative humidity (RH) was recorded hourly during the 4th nymphal instar stage using an automatic recorder (Ondotori TR‐74Ui, T&D Co., Tokyo, Japan). The RH sensor was placed in a container that did not house a locust. Grass leaves were inserted and discarded in the sensor cup as in the other test containers. The mean RH was calculated from data recorded continuously over 3 days.

### 2.6 Recording of Body Color

Body color of locusts was observed 2 or 3 days post‐ecdysis in the 3rd, 4th, and 5th instars. Under white fluorescent light, the test nymphs were classified as having either green or brown body color. The sex of the test individuals was also recorded. However, body color expression did not differ between the two sexes (e.g., 82 males and 77 females obtained from pooled data collected from the 5th instar nymphs in *II_groups 1* to *4*; Figure 2; Fisher's exact test, *p* = 0.097); thus, only the pooled results are shown. In this study, four color morphs were observed: green, brown with faint green, brown, and dark. The first two morphs were categorized as green, and the latter two as brown (Figure S2).

### 2.7 Statistical Analysis

Fisher's exact test was performed to compare two groups using Prism 9 (v. 9.5.1, GraphPad Software Inc., San Diego, CA, USA). *p* < 0.05 was considered statistically significant.

## 3 Results

### 3.1 Effect of Feeding Plant Species Under 23‐h Dark Conditions and Effect of Humidity Under 16‐h Food Availablity Conditions

In this study, we reared *L. migratoria* nymphs in growth chambers under modulated light cycles. Red light‐emitting diodes were used only when handling the insects during the dark period. The number of simultaneously available growth chambers was four or less, and each experiment took a month or more to complete. Consequently, we were concerned that the feeding plant species could affect the results of our study, as the availability of plants changes with season. Accordingly, we first compared the effects of two plant species, *B. catharticus* and *D. glomerata*, which are available during the cold and cool seasons, respectively, as food, on green/brown polyphenism. All experiments in this study were conducted during the cool and cold seasons, as indoor RH is low in Japan. In *S. gregaria* nymphs reared in isolation, exposure to a yellow–green background for just 15 min daily, or a light period of 15 min, has been found to induce a green body color (Tanaka et al. 2012). We also examined whether green morphs appear under a 23‐h dark/1‐h light cycle in *L. migratoria* (Figure 1A). To minimize the effects of visual stimuli other than the yellow–green paper covering the container, all leftover grass and feces were removed immediately before the lights were turned on, and cut grass leaves were provided immediately after the lights were turned off. In this experiment, the average RH in the containers was 70% and 50% when *B. catharticus* and *D. glomerata* leaves were used, respectively (Figure 1B). The proportion of 5th‐instar green nymphs was 50% and 55% when fed on *B. catharticus* and *D. glomerata* leaves, respectively. This indicates that green morphs developed during the 1‐h light period each day, regardless of the plant species used for feeding. In addition, variation in RH values did not influence the proportion of green morphs under these conditions. The proportion of these morphs accounted for approximately half of the total population.

Subsequently, to evaluate the effect of humidity, half of the test nymphs were reared with moist cotton, while the other half were reared with dry cotton. To enhance the occurrence of green morphs, the light period was prolonged to 8 h each day to allow the nymphs to see the yellow–green paper for a longer period (Figure 1C). The test nymphs could not see fresh or leftover grass or feces during the experiment due to darkness. The average RH in the moist cups was 82%, while that in the dry cups was 46% (Figure 1D). Some green morphs appeared at the 3rd instar under moist conditions, whereas only brown morphs were observed at that instar stage under dry conditions. More 4th‐instar green morphs appeared under moist conditions compared with dry conditions (Fisher's exact test, *p* = 0.01). However, the proportion of green morphs at the 5th instar was limited to 64% even when nymphs were reared with moist cotton.

### 3.2 Effect of FAP

Despite the classical model for *L. migratoria* green/brown polyphenism, moist conditions (82% average RH) did not consistently induce green body color. This suggests that humidity is not the only factor essential for regulating green/brown polyphenism in *L. migratoria* nymphs. To explore other factors involved in the appearance of green morphs, we modulated several variables simultaneously: we fixed the light cycle to 16 h of darkness and 8 h of light and changed the timing of food provision (*II_groups 1* to *3* in Figure 2A). The nymphs in *II_group 3* were additionally provided fresh grass just before the dark period to prevent loss of appetite for dried grass leaves. We also created conditions under which the light/dark cycle of *II_group 1* was reversed to investigate whether the FAP is associated with light/dark conditions in the emergence of green morphs (*II_group 4* in Figure 2A). Under these conditions, RH in the cup rose with an increase in the total FAP owing to the continuous release of moisture from the grass leaves. In addition, nymphs were allowed to see grass leaves for 0 (*II_groups 1*), 4 (*II_groups 2*), 8 (*II_groups 3*), and 16 h (*II_group 4*) in Figure 2A, whereas feeding occurred in complete darkness in the experiments shown in Figure 1A,B. As shown in Figure 2B, although no 3rd‐instar green morphs were observed in *II_groups 1* to *3*, the proportions of green morphs at the 4th and 5th instars reached 64% and 94% in *II_group 3*, respectively (Figure 2B), which were significantly higher than the proportions observed in *II_group 1* (Fisher's exact test, *p* < 0.01 in both tests). The average RH in *II_group 1* cups was 30%, while that in *II_group 3* cups was 62% owing to the continuous release of moisture from grass leaves. Thus, longer FAPs, which also result in higher RH and longer time for seeing grass leaves, contributed to the development of green nymphs.

Average RH was slightly higher in *II_group 4* than in *II_group 1* in Figure 2B, whereas the RH value of 47% in *II_group 4* was relatively low compared with conditions prepared by moist cotton (82% in Figure 1D). Several green morphs were observed at the 3rd instar in *II_group 4*, whereas all 3rd instar nymphs were brown in *II_group 1*. The proportion of green morphs at the 4th and 5th instars in *II_group 4* reached 70% and 87%, respectively. These values were significantly higher than those observed in *II_group 1* (Fisher's exact test, *p* < 0.01 in both tests). Although the total FAP was equivalent between *II_group 1* and *4*, the proportion of green morphs differed under the two experimental conditions. Thus, green morphs appeared exclusively under the treatment conditions for *II_groups 3* and *4*.

### 3.3 Effect of Humidity Under 24‐h FAP

Although Figure 1D shows that higher humidity induced green body color, the proportions of green individuals at the 5th instar (51% in a dry cup; 64% in a moist cup) were relatively low compared with those of *II_groups 3* and *4* (94% and 87%, respectively) of Figure 2. Thus, a question arose regarding the importance of humidity in green/brown polyphenism. We speculated that fasting for 8 h has a negative effect on producing green morphs. Accordingly, we prepared test nymphs that were allowed to feed at all times under either dry or moist conditions (Figure 3A). This rearing schedule also created an 8‐h overlap between FAP and light durations, whereas there was no overlap in Figure 1D. Grass was additionally provided to both groups just before the dark period to prevent decreased appetite due to dried grass leaves, particularly in dry cups. The average RH in the moist cups was 78%, while that in the dry cups was 44% (Figure 3B). In this experiment, a green morph at the 3rd instar was observed in a dry cup. However, more green morphs at the 3rd instar were observed in the moist cups (Fisher's exact test, *p* < 0.01). The proportion of green 4th‐instar nymphs in moist cups reached 88%, which was significantly higher than the proportion observed in dry cups (Fisher's exact test, *p* < 0.01). In this experimental design, the nymphs were able to see grass and feces for 8 h. Although it is possible that the fed grass was somewhat discolored during the 8‐h period only in dry cups, the wilting of the grass was not pronounced over such a short period. This result supports the interpretation shown in Figure 1D that high humidity causes brown morphs to turn green. Furthermore, moist conditions can exclusively induce green body color if certain other conditions are met.

### 3.4 Effect of the Total FAP During the Dark Period

Several factors appear to be involved in the induction of green body color, including the total daily FAP. On the other hand, a longer FAP leads to a higher average RH (*II_groups 1* to *3* in Figure 2B). To assess the single effect of the total FAP, nymphs were kept with moist cotton to maintain the RH in the cups and the moisture in grass leaves for 23 h and were fed for 23 (*IV_groups 1*), 20 (*IV_groups 2*), or 16 h (*IV_groups 3*) in the dark (Figure 4A). The nymphs were exposed to a yellow–green background for 1 h daily, as visual information about the surrounding color appears to play an important role in determining locust body color. The average RH was comparable across the three groups (Figure 4B). Despite high humidity (80%), the proportion of green morphs at the 5th instar reached only 33% in *IV_group1*. This low ratio prevented a full comparison of the differences between *IV_group 1* and *IV_group 3* (Fisher's exact test, *p* = 0.12). This indicates that high humidity does not necessarily lead to a high proportion of green morphs. Thus, the single effect of the total FAP could not be assessed in this experimental design.

### 3.5 Effect of Visual Color Information for 8 h During the FAP

The results in Figure 2B suggested that the substrate color of grass or wallpaper affected the body color of nymphs reared in isolation under daily fasting for 8 h. In this experiment, the FAP and light periods coincided. It is possible that the overlapping length of the FAP and light duration is involved in the induction of green morphs. Accordingly, we reared nymphs in isolation in a light cycle of 16‐h darkness/8‐h light under 8‐h fasting. During the FAP, the test nymphs could see the surrounding color for 0 (*V_groups 1*), 4 (*V_groups 2*), and 8 h (*V_groups 3*) (Figure 5A). Even at the 5th instar, the proportion of green morphs was low across all groups (Figure 5B). While fasting for 8 h may decrease the proportion of green nymphs, 16‐h FAP during the light period canceled out the fasting effect, as shown in Figure 2B. Thus, 8‐h FAP during the light period may not be sufficient to overcome the fasting effect.

### 3.6 Effect of Visual Color Information for 12 h During the FAP

To extend the FAP to 12 h during the light period, nymphs reared in isolation and fasted for 8 h were kept in a 12‐h darkness/12‐h light cycle. During the FAP, the test nymphs could see the surrounding color for 12 (*VI_groups 1*), 8 (*VI_groups 2*), and 4 h (*VI_groups 3*) (Figure 6A). Some green morphs appeared at the 4th instar only in *VI_groups 1* and *2* (Figure 6B). The proportion of green morphs at the 5th instar was notably different among the three groups. The difference between *VI_groups 1* and *3* at the 5th instar was significant (Fisher's exact test, *p* < 0.01). Thus, 12‐h FAP during the light period was able to overcome the fasting effect on inducing green morphs.

## 4 Discussion

Green/brown polyphenism has been observed in many acridoids, tettigonioids, mantoids, phasmids, cicadids, and lepidopterans (Rowell 1972). In the widely accepted hierarchical model for locust body color, humidity is placed at the second level of regulation (Pener 1991; Pener and Simpson 2009). In contrast, *S. gregaria* nymphs, unable to see grass under dark conditions, do not respond to humidity, suggesting that humidity has little effect on green/brown polyphenism in this species (Tanaka et al. 2010, 2012). A similar experiment for *L. migratoria* conducted in the present study revealed that humidity increased the occurrence of green morphs under a 16/8 h dark/light cycle (Figures 1D and 3B). However, the regulation of green/brown polyphenism by humidity is complex. Under a 23‐h dark/1‐h light cycle, the proportion of green morphs at the 5th instar was always below 60%, despite high humidity (over 70% average RH) (Figures 1B and 4B). This suggests that *L. migratoria* nymphs are not sensitive to humidity regarding green/brown color polyphenism under this light cycle. Visual information is strongly associated with this polyphenism. High humidity may effectively induce green morphs when light is provided for 8 h or more per day (Figure 3B), which is the natural condition in most *L. migratoria* habitats.

The level of moisture in grass was closely associated with the humidity in the cups because grass leaves continuously released moisture during the experiment. Previous studies have proposed that humidity and the level of moisture in the diet are the most important environmental factors for green/brown color polyphenism in many grasshoppers, which is consistent with the hierarchical model (Dearn 1990; Rowell 1972). In the present study, we compared the effects of two feeding plants, *B. catharticus* and *D. glomerata*, on polyphenism. *B. catharticus* was moister than *D. glomerata* during the season in which this study was conducted, resulting in higher humidity in the cup where *B. catharticus* was present. According to our data, green/brown polyphenism was not affected by the level of moisture in grass (Figure 1B) but might have been influenced by a regular short light period of 1 h, as discussed above.

Another factor contributing to green/brown color polyphenism in *L. migratoria* was overlapping periods of food availability and light. When test nymphs were fasted for 8 h each day, individuals exposed to a 12‐h overlapping period of food availability and lighting showed a higher proportion of green morphs compared with those exposed for only 4 h (*VI_groups 1* and *3* in Figure 6B). In contrast, nymphs in both groups could see the yellow–green wallpaper for 12 h in this experiment. The difference between the two groups was possibly caused by visual information regarding the color of the feeding grass. During feeding, nymphs may observe grass color, which affects their body color. Alternatively, the color of the grass may have a greater influence on polyphenism than yellow–green wallpaper. After the cut grass leaves were inserted into the rearing cup, the nymphs gradually ate them at intervals (Figure S3A,B) (Chapman 1998). Accordingly, these nymphs may observe grass color during the intervals of digestion. If grass color was the dominant factor affecting polyphenism, the proportion of green morphs would fluctuate depending on the timing of the experiments, as grass colors vary somewhat with seasons and plant species. Tanaka et al. (2012) observed that the yellowing of grass over time had little effect on green/brown polyphenism in *S. gregaria* nymphs reared in isolation. This suggests that green color in grass is not essential for producing green morphs in *S. gregaria*. Another possibility involves neuronal interactions between feeding and vision, suggesting a complex integration of sensory inputs. The mouthparts and salivary glands are regulated by the suboesophageal ganglion (SOG), which interconnects with the optic lobes (OL) (Chapman 1998; Uvarov 1966). Both nerves belong to the central nervous system and may cooperate to integrate visual sensitivity and feeding behavior through neuropeptides in *S. gregaria* (Dillen et al. 2014). Activation of SOG during feeding may stimulate the OL, resulting in increased sensitivity to the substrate color of the habitat. Further studies are necessary to determine why the overlap in food availability and light periods affects green/brown color polyphenism in this species.

Based on the results of the present study, we cannot conclude whether the total daily FAP is involved in green/brown polyphenism in *L. migratroria*. This uncertainty arises because an increase in the FAP was accompanied by high humidity and visual stimuli from the grass. Although we attempted to isolate the effect of the total FAP by controlling other factors (Figure 4), the results were inconclusive. As discussed earlier, the proportion of green morphs at the 23‐h dark/1‐h light cycle was always low. In contrast, nymphs fasted for 8 h demonstrated lower proportions of green individuals (Figures 1, 2, 3, 5, and 6B) only when green‐inducing factors were not present. We observed that 5th instar nymphs reared in cups consumed grass for an average of 11.9 min at intervals of 87.4 min (Figure 3A,B), suggesting that fasting for 8 h suppressed feeding in test nymphs. The green pigments in most green plants are chlorophylls, whereas the visible green coloration in locusts is composed of yellow and blue pigments (Matile et al. 1999; Pener 1991). Thus, the decreased intake of chlorophylls due to daily fasting is unlikely to be directly associated with brown body color. The effect of the total FAP should be further analyzed in future studies.

A previous study reported that varying the temperature and substrate color in rearing containers can change the proportion of green morphs in *S. gregaria* (Tanaka et al. 2012, 2016). The temperature and substrate color in the rearing cups stimulated the expression of the yellow protein of the takeout family gene *YPT* in *S. gregaria* (Sugahara and Tanaka 2018, 2019). Yellow and blue pigments are involved in locust green coloration (Pener 1991). We conducted the present study at a constant temperature 30°C in growth chambers, using rearing cups covered with yellow–green paper. These two factors would further complicate the regulation of green/brown polyphenism in *L. migratoria* nymphs.

The coloration of *L. migratoria* nymphs is associated with habitats that exhibit seasonal changes and local spatial variations (see Frontispiece VI, Tanaka 2021). Is there an ecological significance in regulating green/brown color polyphenism in *L. migratoria* through humidity and overlapping FAP and light periods? In regions with a dry climate, a moist atmosphere becomes a cue to manifest green body color during and after the rainy season (Rowell 1972). This resultant cryptic coloration among green vegetation may contribute to a selective advantage during the season. In contrast, in wet temperate zones, the green body color does not match withered grass, exposed ground, and locally burned grassland (see Frontispiece VI, Tanaka 2021). It is likely that brown morphs of *L. migratoria* often remain in these fields, which correlates with a cryptic background during the daylight period, as brown individuals are frequently found in such fields in wet temperate zones. However, green grass is less available as food on exposed ground and locally burned grasslands. This raises the following question: when do brown morphs feed on grass? Although many grasshopper species do not feed in total darkness, *Acrida* and *Locusta* do feed under these conditions (Rowell 1972). Consistent with the feeding habits of *Locusta*, *L. migratoria* nymphs fed on grass during dark periods. If brown nymphs of *L. migratoria* primarily feed at night, this behavioral trend could result in their body color becoming brown because of the short overlap between the FAP and light periods. Further studies are needed to examine the feeding patterns of green and brown morphs under light and dark cycles to better understand their ecological adaptations.

## Author Contributions

**Keiryu Hirota:** investigation, conceptualization, data curation, methodology. **Ryo Watanabe:** investigation. **Ryohei Sugahara:** investigation, supervision, data curation, writing–original draft, project administration, conceptualization, writing–review and editing.

## Conflicts of Interest

The authors declare no conflicts of interest.

## Supporting information

Supporting information.

Supporting information.

Supporting information.

Supporting information.

Supporting information.

## Data Availability

The data that support the findings of this study are available from the corresponding author upon reasonable request.
