# Supplementary material for: The Effect of Humidity and the Role of Visual Cues During Feeding on Green/Brown Color Polyphenism in Locusta migratoria
Source: Arch Insect Biochem Physiol. 2025 Feb 25;118(2):e70044. doi: 10.1002/arch.70044 (PMC11861565; doi:10.1002/arch.70044)
Supplement: Supplementary file 1 — Supporting information. [file ARCH-118-e70044-s001.docx]

**Supplementary text**

**Supplementary Materials and Methods**

*Mortality of nymphs reared in isolation and under daily fasting for 8 h*

Hatchlings and newly ecdysed 2nd instar nymphs (day 0) were transferred to transparent plastic cups (bottom diameter, 8 cm; lid diameter, 9 cm; height, 2.5 cm) covered with a perforated lid with four holes for ventilation, as described in the Materials and Methods section of the main text. The nymphs were reared without moist cotton according to the schedule shown in Fig. 1C. Nymphal mortality was recorded at the 5th instar.

*Monitoring of feeding behavior*

Four 5th instar nymphs were reared individually in cups. Their feeding behavior was automatically recorded using a network camera (Atom Cam 2, ATOM Tech Inc., China) during the light period. The intervals and durations of feeding were manually calculated from the video footage.

**Supplementary Legends**

**Supplementary Fig. 1.** Tools and equipment used for rearing insects. (A) A perforated lid was created for locust rearing. (B) Rearing containers were kept in a growth chamber. This was photographed with the growth chamber lights off and the room lights on. (C) This was photographed with the fluorescent lights off and a red light-emitting diode on in the growth chamber, with the room lights off. (D) A moist cotton ball was wrapped in silicon-coated paper to keep the yellow–green paper inside dry.

**Supplementary Fig. 2.** Photographs depicting the green or brown body colors of 5th instar nymphs. Sixteen individuals, including both males and females with either green or brown coloration, were haphazardly selected from the nymphs that emerged in the present study. Some penultimate instar females, which have short wing pads (indicated by red arrowheads), were categorized as 5th instar nymphs, as described in section 2.1 of the Materials and methods.

**Supplementary Fig. 3.** The intervals (A) and durations (B) of grass eating were plotted for each nymph at the 5th instar. The bars represent average values.

**Supplementary Table 1.** Mortality of *L. migratoria* nymphs under daily fasting for 8 h
