# Supplementary figures and images for: The Effect of Humidity and the Role of Visual Cues During Feeding on Green/Brown Color Polyphenism in Locusta migratoria
Source: Arch Insect Biochem Physiol. 2025 Feb 25;118(2):e70044. doi: 10.1002/arch.70044 (PMC11861565; doi:10.1002/arch.70044)

Supplementary Fig. 1

A

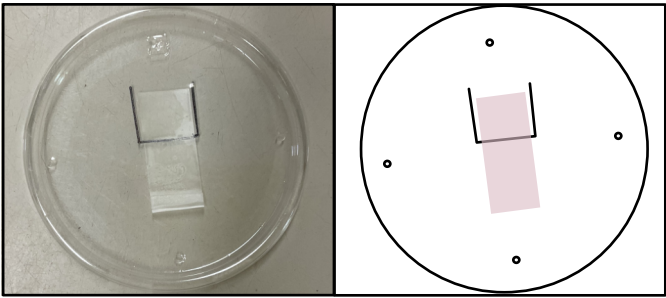

- Air hole
- └ Slit for grass insertion
- Clear vinyl tape

B

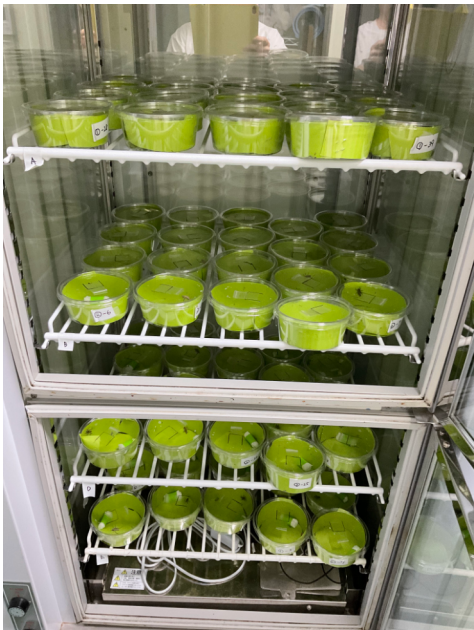

C

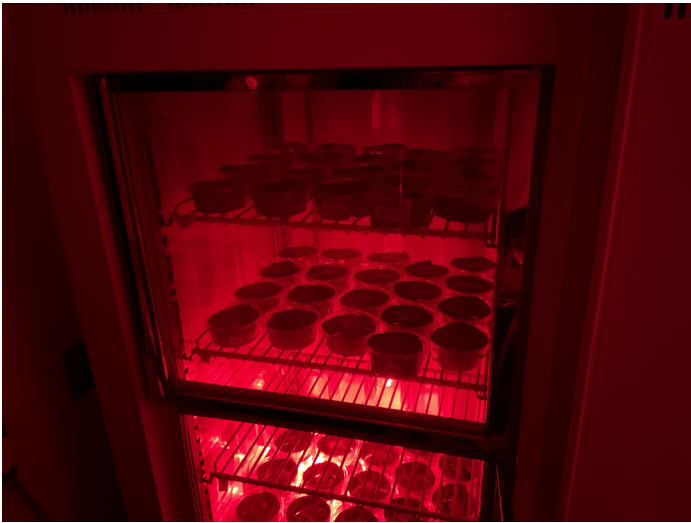

D

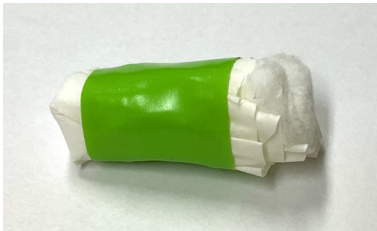

Supplement: Supplementary file 2 — Supporting information. [file ARCH-118-e70044-s002.pdf]

Supplementary Fig. 2

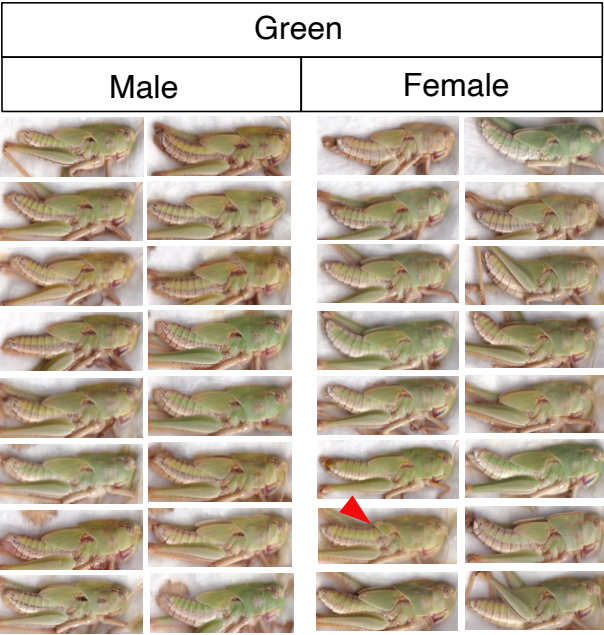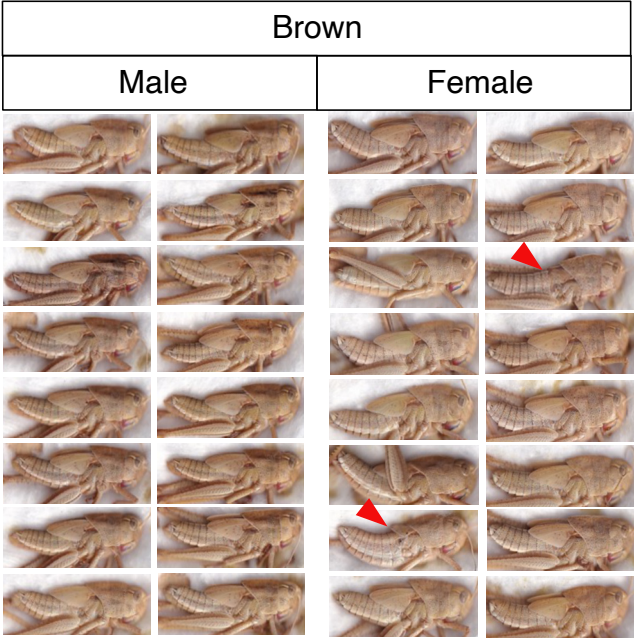

Supplement: Supplementary file 3 — Supporting information. [file ARCH-118-e70044-s005.pdf]

Supplementary Fig. 3

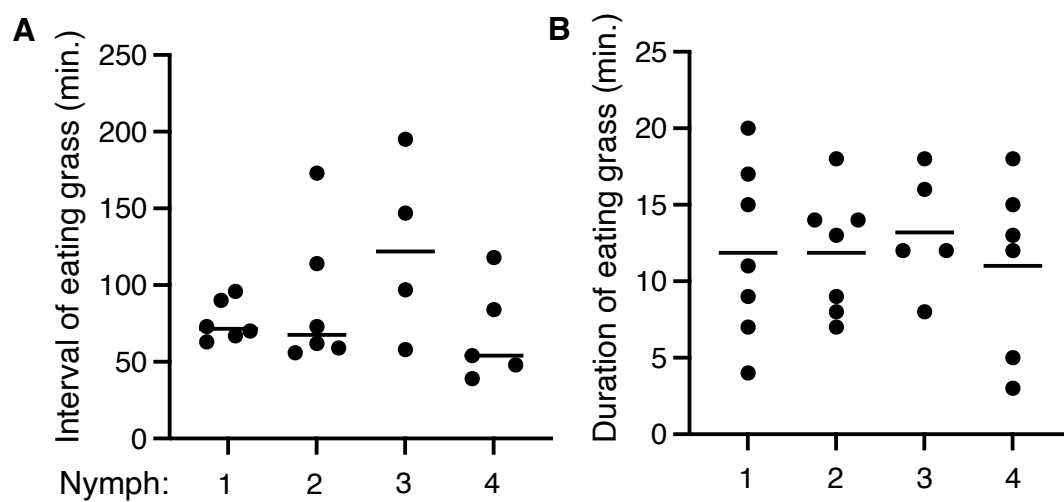

Supplement: Supplementary file 4 — Supporting information. [file ARCH-118-e70044-s004.pdf]
