# Supplementary material for: The Effect of Humidity and the Role of Visual Cues During Feeding on Green/Brown Color Polyphenism in Locusta migratoria
Source: Arch Insect Biochem Physiol. 2025 Feb 25;118(2):e70044. doi: 10.1002/arch.70044 (PMC11861565; doi:10.1002/arch.70044)
Supplement: Supplementary file 5 — Supporting information. [file ARCH-118-e70044-s003.pdf]

Supplementary Table 1 Mortality of *L. migratoria* nymphs under daily fasting for 8 hours.

Subjected nymph	Mortality	No. of test nymph
1st instar nymph	77%	40
2nd instar nymph	0%	20
